# Toxic Epidermal Necrolysis Induced by Doxycycline: A Case Report and Literature Review

**DOI:** 10.7759/cureus.72098

**Published:** 2024-10-22

**Authors:** Nicolas Sandakly, Georgio El Koubayati, Lea Issa, Rim M Abdallah, Selim Nasser, Fady Haddad

**Affiliations:** 1 Department of Internal Medicine, Lebanese University Faculty of Medicine, Beirut, LBN; 2 Department of Internal Medicine and Clinical Immunology, Lebanese University Faculty of Medicine, Beirut, LBN; 3 Department of Internal Medicine and Clinical Immunology, Lebanese Hospital Geitaoui - University Medical Center, Beirut, LBN; 4 Department of Pathology, Lebanese American University Medical Center, Beirut, LBN

**Keywords:** case presentation, doxycycline, drug eruptions, drug-related side effects and adverse reactions, toxic epidermal necrolysis (ten)

## Abstract

Lyell syndrome, also known as toxic epidermal necrolysis (TEN), is a rare but life-threatening skin condition, often triggered by certain medications. Antiepileptics, allopurinol, and some nonsteroidal anti-inflammatory drugs are the most common causes. Some antibiotics are also common culprits, although tetracyclines are rarely linked to this condition. We hereby report a case of TEN induced by doxycycline in an 18-year-old girl who was taking the drug for the treatment of acne.

## Introduction

Toxic epidermal necrolysis (TEN), also known as Lyell syndrome, is a severe, life-threatening skin condition that predominantly results from adverse drug reactions. It is characterized by widespread necrosis and detachment of the skin and mucous membranes, affecting more than 30% of the body surface area, making it one of the most extreme forms of drug-induced skin reactions [1]. TEN shares a clinical spectrum with Stevens-Johnson syndrome (SJS), and both are considered severe cutaneous adverse reactions. The primary difference between TEN and SJS is the extent of skin involvement: SJS involves less than 10% of the body surface area, while TEN involves more than 30%, with an overlap syndrome involving between 10% and 30% of the body surface area [2].

TEN can begin with flu-like symptoms, including fever, sore throat, and malaise, before progressing rapidly to painful red or purplish rashes that lead to skin detachment. This extensive skin loss can result in serious complications, including infections, dehydration, and multiorgan failure [3]. The condition demands immediate medical attention, typically in burn units, due to the high risk of mortality (up to 30%-50% depending on the extent of skin involvement). Early withdrawal of the suspected drug and intensive supportive care are crucial for survival [4].

Drugs are the primary trigger for TEN, with antiepileptic medications like phenytoin, carbamazepine, and lamotrigine being significant culprits, as noted in the European Severe Cutaneous Adverse Reaction study [5]. The literature reports only three times that doxycycline, a tetracycline antibiotic, is involved in the development of TEN [6-8]. To this, we add our case of an 18-year-old girl who was taking the antibiotic to treat her acne.

## Case presentation

An 18-year-old female patient presented to the emergency department of the Lebanese Hospital Geitaoui University Medical Center (LHG-UMC), Beirut, Lebanon, presenting with a diffuse mucocutaneous eruption that persisted for a duration of two weeks. The patient was previously healthy. Two weeks before the eruption, the patient was started on doxycycline 100 mg daily for the treatment of acne vulgaris. Following a two-week initiation of the medication, the patient reported experiencing a new onset of malaise, accompanied by a burning sensation in both her eyes and genital area. Additionally, she noted the emergence of a rash that began on her face and chest and rapidly spread to other areas of her body. The patient described the rash as pruritic. She denied taking illicit drugs, herbal supplements, or any over-the-counter medications besides doxycycline.

**Figure 1:**
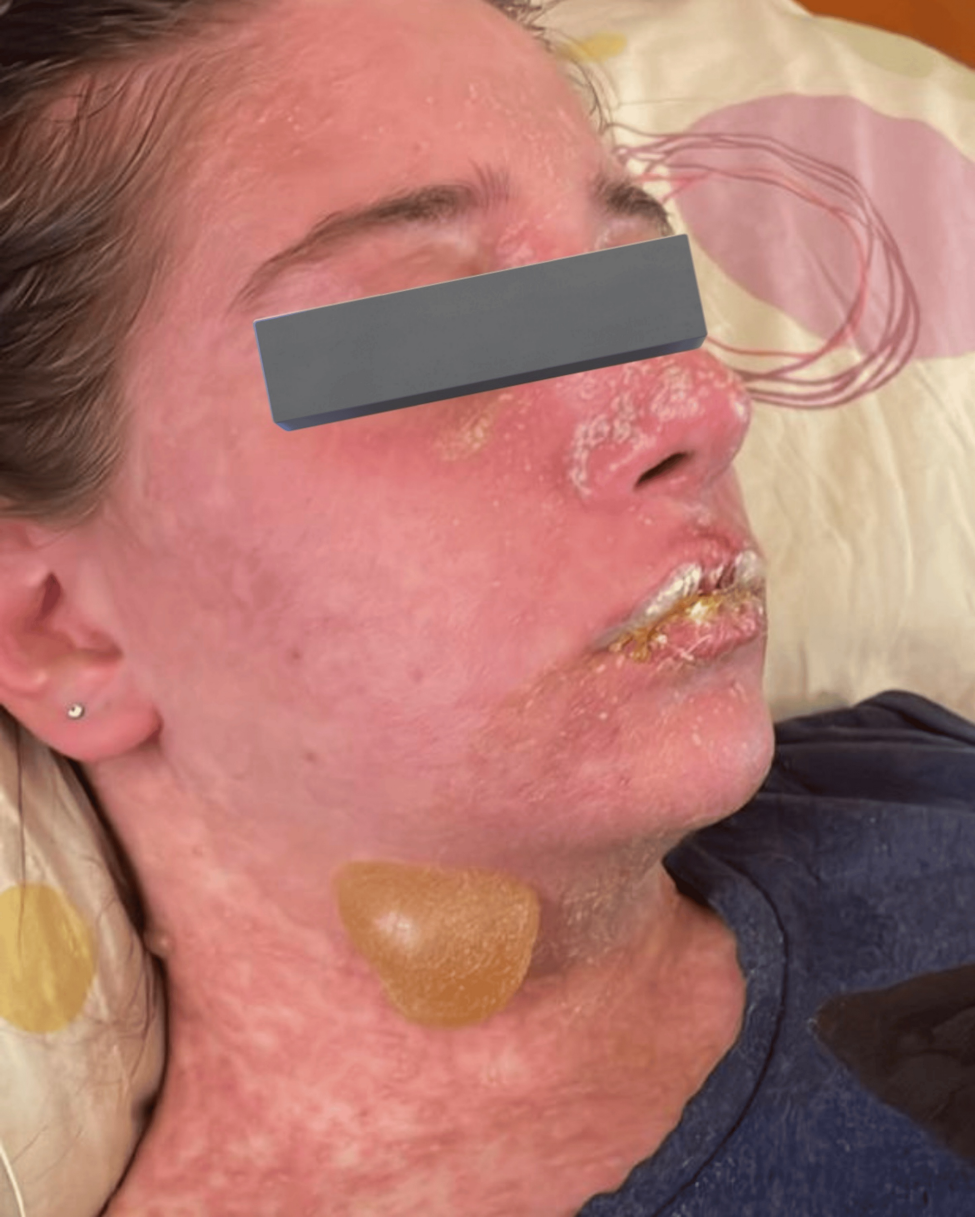
Diffuse tender dusky erythema with macules over the face and lips, along with flaccid bullae over the neck sparing of the scalp

**Figure 2:**
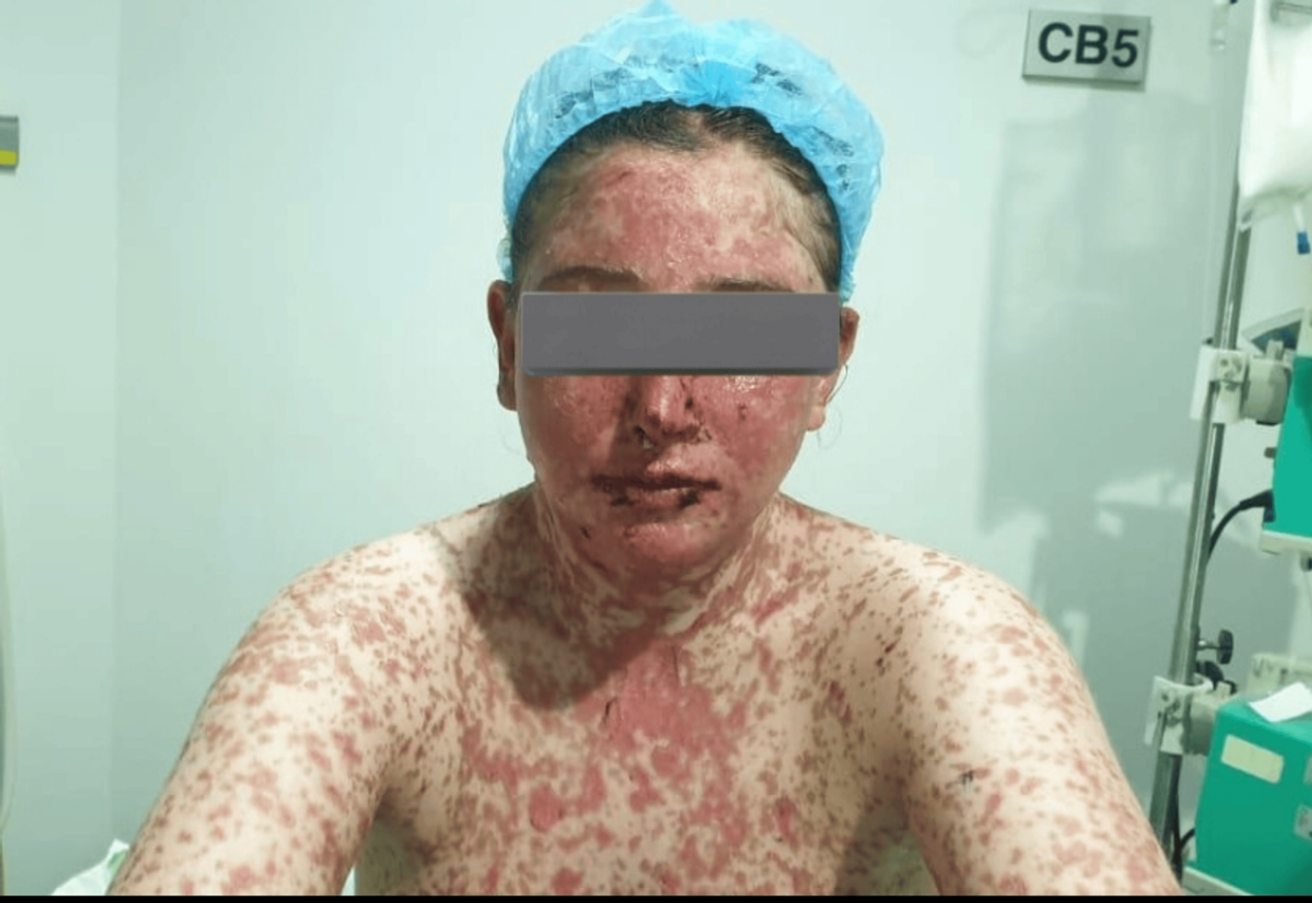
Diffuse tender dusky erythema with macules over the face, limbs, and chest

**Figure 3:**
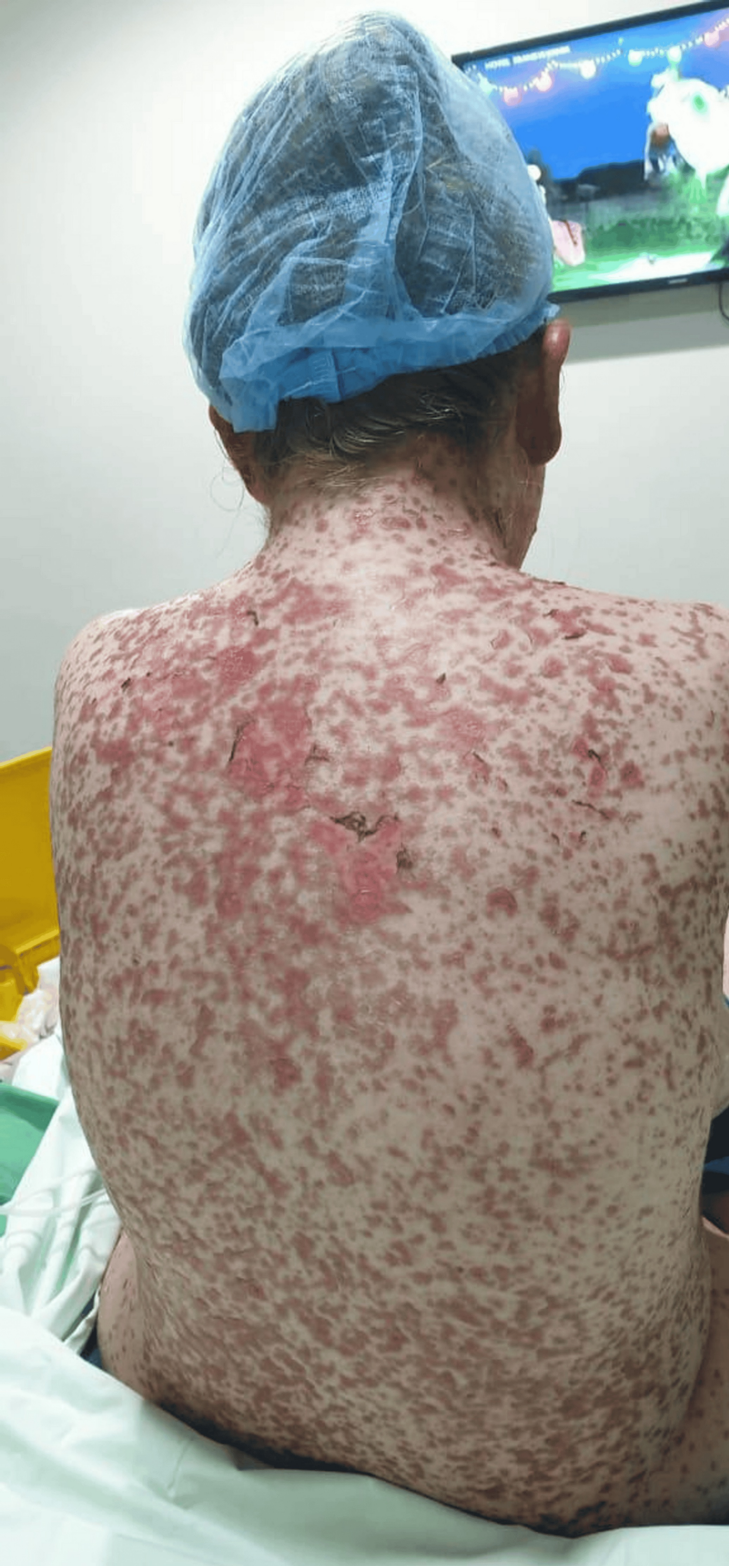
Erythema with macules and skin detachment over the back of the patient. The affected total body surface area was estimated at 80% with 12% skin detachment

On admission, the patient was afebrile and tachycardic with a heart rate of 124 beats per minute, blood pressure of 104/69 mmHg, oxygen saturation of 99% on room air, and respiratory rate of 22 breaths per minute. On physical examination, the affected total body surface area was estimated at 80% with 12% skin detachment. She had diffuse tender dusky erythema with macules over the limbs, chest, neck, and back, and flaccid bullae over the neck, sparing the scalp. Nikolsky sign was positive. In addition, she had involvement of eyes, oronasopharyngeal, and anogenital mucosa (Figures 1-3).

Initial laboratory investigation revealed a hemoglobin of 12 g/dL (reference range of 12-16 g/dL), white blood cell counts of 8,050 (reference range of 4,800-10,800/mm^3^), C-reactive protein of 52 mg/L (reference range <6 mg/L), and a procalcitonin level of 0.06. Liver and kidney function tests were found to be within the reference range. A clinical diagnosis of TEN was made. Her severity of illness score for toxic epidermal necrolysis (SCORTEN), a prognostic score estimating the risk of death in the acute phase, was calculated at 2, placing our patient at a 12% mortality risk (Table 1).

**Table 1:** SCORTEN Patient's SCORTEN: 2 Predicted mortality: 12% SCORTEN: score for toxic epidermal necrolysis

Prognostic factor	Value	Points
Age (years)	>40	0 (18)
Malignancy	Present	0 (No malignancy)
Skin lesion	>10% of total body surface area	1 (80%)
Serum urea (mg/dL)	>28	0 (15)
Serum bicarbonate (mEq/L)	<20	0 (23)
Blood glucose (mg/dL)	<252	0 (273)
Heart rate (bpm)	>120	1 (124)

The patient was admitted to the burn center of the LHG-UMC, where she was isolated and received supportive care with fluid and electrolyte replenishment. Dressings were changed every 48 hours using only petroleum jelly and paraffin gauze. Doxycycline was withdrawn, and the patient was started on intravenous methylprednisolone 250 mg per day, clindamycin 600 mg every eight hours, and cyclosporine (3 mg/kg body weight per day). She also received eye drops of tobramycin and dexamethasone. Parenteral nutrition was started until oral mucosal lesions subsided. She had no pulmonary, gastrointestinal, or renal involvement. Biopsies taken on admission from her left arm and right thigh skin showed severe interface dermatitis with extensive keratinocyte necrosis, consistent with TEN (Figure 3).

**Figure 4:**
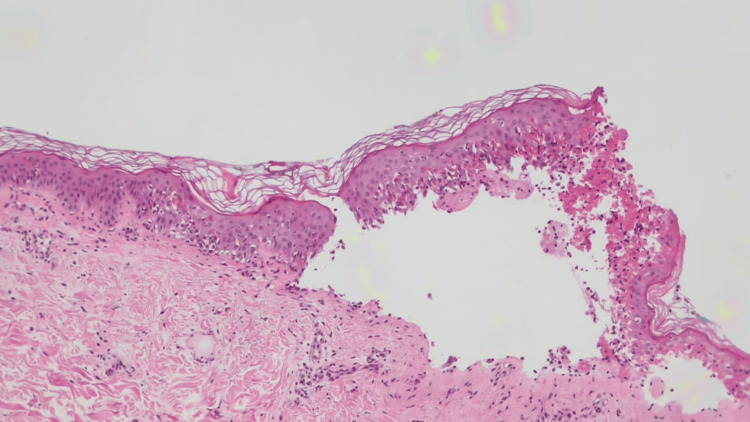
Histopathological findings of skin biopsy. Subepidermal bulla formation with apoptotic keratinocytes and focal full epidermal necrosis. Minimal dermal inflammation (hematoxylin and eosin stain 100×)

**Figure 5:**
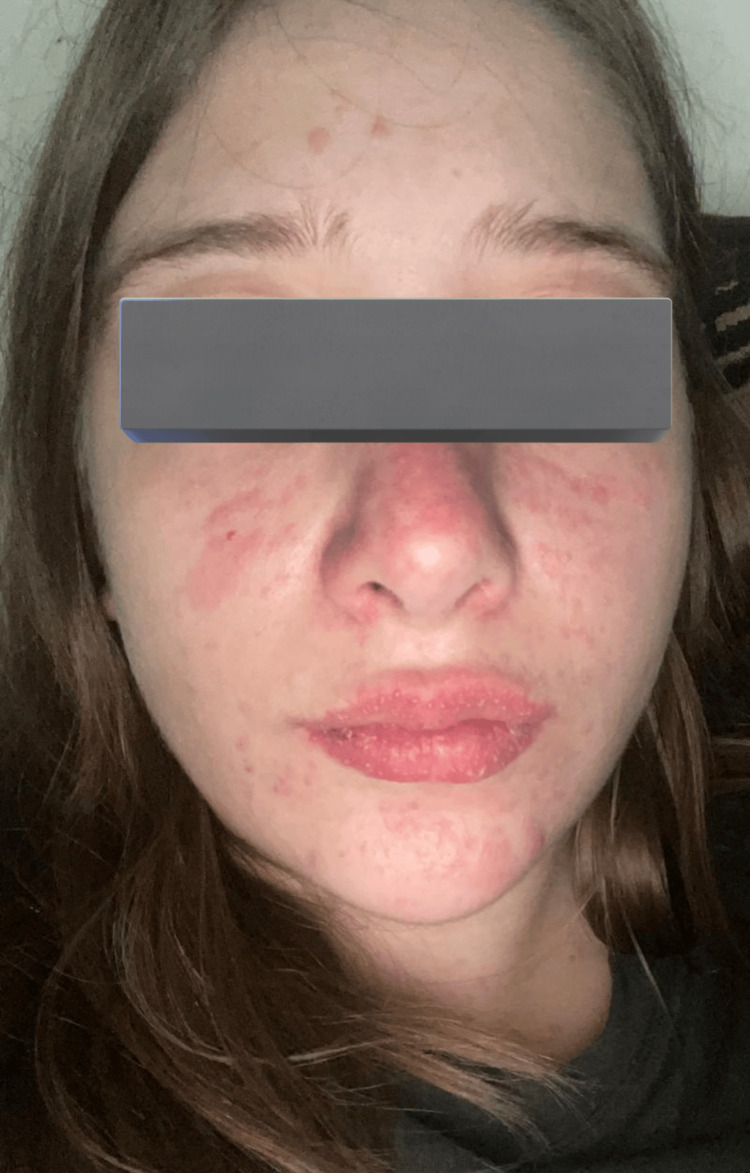
Facial skin lesions two weeks after discharge from hospital

**Figure 6:**
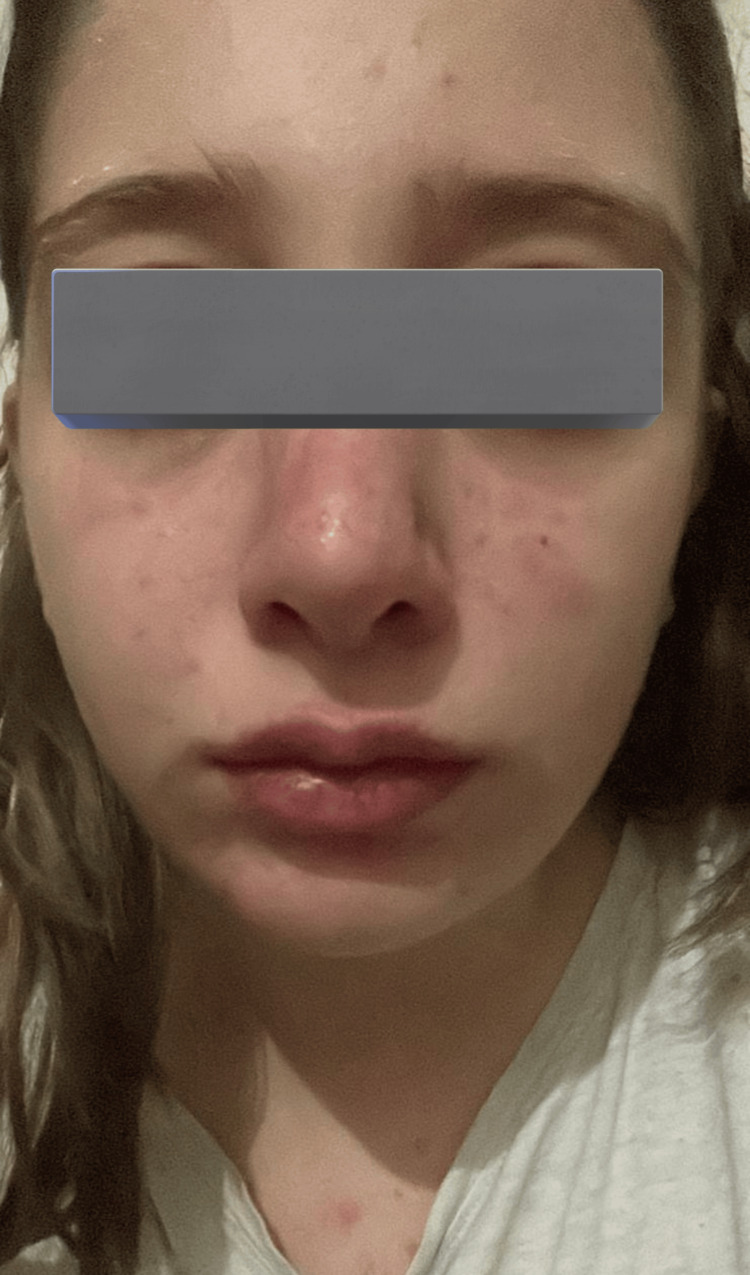
Resolution of skin lesions four weeks after discharge from the hospital

Mucocutaneous lesions gradually improved (Figures 5, 6), and her hospital stay was uneventful. After 20 days of hospitalization, she was discharged home. She was educated about the necessity of avoiding doxycycline and the risk of reexposure.

## Discussion

TEN is the most severe form of drug-induced skin reactions. It is a rare immune-mediated mucocutaneous reaction characterized by skin detachment and necrosis with more than 30% involvement of the body surface area [1]. Antiepileptic drugs, such as phenytoin, carbamazepine, lamotrigine, allopurinol, and oxicam nonsteroidal anti-inflammatory drugs, are classified as high-risk drugs according to the European Severe Cutaneous Adverse Reaction study surveillance of medications [5]. Antibiotics, mainly sulfa drugs and aminopenicillin, are also commonly implicated drugs. However, tetracyclines are rarely associated with TEN. The literature review revealed very few cases of doxycycline-induced SJS, which is considered a variant of the same disease process as TEN, with less than 10% skin involvement and even fewer reported cases of TEN. A thorough search of the PubMed database using the keywords "Toxic epidermal necrolysis" and "doxycycline" revealed only three reported cases from 1974 to 2023, while a limited number of cases of SJS and TEN have been described after tetracycline intake [6-8]. Adelman et al. [9] reported the case of a 39-year-old female patient who developed blistering disease two weeks after completing a one-week course of doxycycline for her upper respiratory tract infection. The patient had a 50% body surface area involvement, and complete reepithelialization was observed during a two-week follow-up. Similarly, Kulkarni et al. [10] reported the case of an 85-year-old female patient who developed TEN eight months after reexposure to doxycycline and presented with a 40% epidermal sloughing Nikolsky-positive skin lesion. Kuster et al. [11] described the case of a 33-year-old female patient with lupus who developed onychomadesis, painful inflammatory neuropathy in the fingers and hands, and severe chronic keratitis and conjunctivitis as a complication of her doxycycline-induced TEN.

Our patient's skin eruption started two weeks after taking doxycycline to treat her acne. Upon admission to our burn center, her skin eruption covered about 80% of her body surface area with up to 10% epidermal detachment. According to Naranjo's adverse drug reaction algorithm (Table 2), a score of 7 was obtained. TEN, probably caused by doxycycline, was considered, which was further supported by the fact that no other medication had been introduced in the past three months. Additionally, no over-the-counter medications or herbal products have been ingested recently. The patient had neither received any recent immunization nor had any recent upper respiratory tract infection. Histopathology results confirmed the diagnosis of TEN.

**Table 2:** Naranjo's score Naranjo's score: ≥9: definite, 5-8: probable, 1-4: possible, ≤0: doubtful Total score of 7 in our patient, indicating probable adverse drug reaction

Question	Yes	No	Do not know	Results
1. Are there previous conclusive reports on this reaction?	+1	0	0	Yes
2. Did the adverse event appear after the suspected drug was administered?	+2	-1	0	Yes
3. Did the adverse event improve when the drug was discontinued or a specific antagonist was administered?	+1	0	0	Yes
4. Did the adverse event reappear when the drug was readministered?	+2	-1	0	Do not know
5. Are there alternative causes that could, on their own, have caused the reaction?	-1	+2	0	No
6. Did the reaction reappear when a placebo was given?	-1	+1	0	Do not know
7. Was the drug detected in blood or other fluids in concentrations known to be toxic?	+1	0	0	Do not know
8. Was the reaction more severe when the dose was increased or less severe when the dose was decreased?	+1	0	0	Yes
9. Did the patient have a similar reaction to the same or similar drugs in any previous exposure?	+1	0	0	No
10. Was the adverse event confirmed by any objective evidence?	+1	0	0	No
Total score	-	-	-	7

A severity-of-illness score for TEN (SCORTEN) combining seven independent risk factors (age ≥40 years, heart rate ≥120 per minute, history of cancer/hematological malignancies, involved body surface area >10%, serum urea level >10 mmol/L, serum bicarbonate level <20 mmol/L, and serum glucose level >14 mmol/L) is one of the most prognostic scores used for TEN patients [12]. However, recent studies have indicated that accuracy may be limited. In a retrospective study, which included 24 TEN patients, assessing the accuracy of SCORTEN, none of the variables included in SCORTEN were a good prognostic factor. The sensitivity of this cohort was estimated at 100%, with a specificity of only 23.81%. Furthermore, the estimated death was 41.9%, whereas the actual death rate was 12.5%, although this decrease in mortality could be attributed to the combined treatment protocol of plasmapheresis and intravenous immunoglobulin [13]. A meta-analysis by Torres-Navarro et al. [14] evaluating the efficacy of various treatment modalities for TEN using the SCORTEN score concluded that cyclosporine and a combination of immunoglobulins with corticosteroids were linked to a lower mortality rate than predicted by SCORTEN.

## Conclusions

This case report describes a rare occurrence of doxycycline-induced TEN in an 18-year-old female, underscoring the potential severity of drug-induced skin reactions, even with medications not commonly associated with such outcomes. The onset of TEN occurred two weeks after starting doxycycline for acne vulgaris, a drug rarely implicated in this severe mucocutaneous reaction. The patient was treated effectively with cyclosporine, corticosteroids, and supportive care, resulting in a favorable outcome and full recovery after 20 days of hospitalization. This case emphasizes the need to recognize doxycycline as a possible cause for severe drug reactions, careful consideration when prescribing, and prompt withdrawal of the offending drug upon diagnosis.
